# Field-parallel six-sample microfluidic detection of plant viruses via raffinose-assisted one-pot LAMP-CRISPR/Cas12b

**DOI:** 10.1016/j.jare.2025.08.030

**Published:** 2025-08-22

**Authors:** Jiahui Wang, Wenqing Yang, Jingjing Fang, Henghao Xu, Jianyao Li, Yibin Zhang, Qiaolan Liang, Bingliang Xu, Huixia Li, Erbo Niu

**Affiliations:** College of Plant Protection, Gansu Agricultural University, Lanzhou 730070, China

**Keywords:** Plant virus, LAMP-CRISPR/Cas12b, Raffinose, Microfluidic, In-field application

## Abstract

**Introduction:**

Plant viral diseases threaten food security globally. Rapid, accurate and early detection of viral pathogens is critical for proactive surveillance and effective control.

**Objectives:**

This study aimed to develop a platform for the diagnosis of plant viruses in the field.

**Methods:**

We developed an ALERT platform (AapCas12b coupled RT-LAMP Extraction-field RNA testing) that integrates RT-LAMP and CRISPR/Cas12b-based lateral flow system for the diagnosis of plant viruses. The portable metal incubator, coupled with the integrated microfluidic chip, enables parallel detection of six test samples in the field. Additionally, a raffinose additive solution effectively achieved the phase separation between the LAMP and CRISPR/Cas12b system through promoting dynamic diffusion of LAMP products amplicons.

**Results:**

The ALERT platform exhibited comparable sensitivity to conventional RT-PCR, and field detection showed 100% diagnostic agreement with RT-PCR.

**Conclusions:**

This platform exhibits outstanding performance with high sensitivity and portability, and has great potential for rapid plant virus detection, especially in environments with resource-limited settings.

## Highlights

• ALERT integrates LAMP and CRISPR/Cas12b in a microfluidic chip for simultaneous detection of six plant-virus samples.
• Raffinose-assisted one-pot design separates LAMP and Cas12b reactions, lowering contamination risk.
• Integrated nucleic acid extraction, microfluidic chip, and lateral flow enable field detection in resource-limited settings.
• ALERT achieves 10^2^ fg/µL detection within 1 h, with sensitivity comparable to lab-based RT-PCR.

## Graphical abstract

**Figure:**
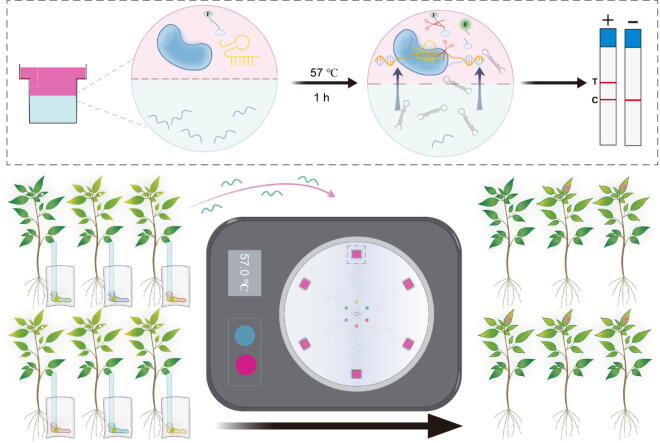

## Introduction

Rapid, specific and highly sensitive identification is critical to control and prediction of plant disease particularly in the viral diseases because of lack of commercially available chemistries against plant viruses [1]. Many of viral diseases have not been early diagnosis and precise identification timely at the seedling stage, leading to pandemic of plant viruses and then outbreaks of diseases. It is also essential to conduct large-scale virus detection in the field. Traditional plant virus detection methods include polymerase chain reaction (PCR), quantitative reverse transcription PCR (qRT-PCR), which are laborious, time consuming, and require specialized laboratory equipment. In this context, the development of affordable, sensitive, rapid and field-deployable diagnostic tests is an urgent need for plant viral diseases prediction and control.

Isothermal nucleic acid amplification assays, such as loop-mediated isothermal amplification (LAMP), recombinase polymerase amplification (RPA), have been developed as the attractive alternative to conventional method for the detection of plant viruses because of its simplicity, low cost, and sensitivity [[2], [3], [4]]. However, the presence of potential false positives and lower accuracy has posed a significant challenge [4,5]. Recently, CRISPR/Cas (clustered regularly interspaced short palindromic repeats/CRISPR-associated) systems, as excellent genome editing tools, has emerged as promising diagnostic techniques [2,6,7]. Cas12, Cas13 and Cas14 are major Cas proteins involved in nucleic acid detection based on their *trans*-cleavage activity upon specific target DNA or RNA recognition [[6], [7], [8], [9]]. CRISPR/Cas12 activated by CRISPR RNAs (crRNAs) can specifically recognize double-stranded DNA (dsDNA), followed *trans*-cleavage nearby single-stranded DNA (ssDNA) [10,11]. By combining with nucleic acid pre-amplification procedures such as RPA (recombinase polymerase amplification) or RT-LAMP (reverse transcription loop-mediated isothermal amplification) [12], CRISPR/Cas12 systems have been developed various detection platforms, such as Cas12a-based DETECTR (DNA Endonuclease Targeted CRISPR Trans Reporter) [8], Cas12a-based HOLMESv1 (a one-HOur Low-cost Multipurpose highly Efficient System), Cas12b-based HOLMESv2, Cas12b-based DNA detection (CDetection) [13] and Cas12a-based visual detection with RPA [14]. However, many of these assays employ a two-pot detection strategy, thus it complicates the process and increase the risk of aerosol contamination [5,12]. To address these challenges, Zhao et al. combined an all-in-one reverse-transcription recombinase-aided amplification (RT-RAA) with a Cas12a-based lateral flow assay in one mixture to develop a suitable strategy for rapid and accurate field detection of four tobamoviruses, while detection efficiency was drastically compromised due to poor biocompatibility [4,7].

Microfluidics has been explored as a powerful tool for diseases detection due to its evident advantages, such as miniaturization, cost-effectiveness, portability, quick and accurate response [15,16]. In addition, microfluidic platforms enable simultaneous identification of multiple targets through using spatial separation of detection sites [5,17,18]. For instance, a Flexible, Robust, Equipment-free Microfluidic (FREM) platform was developed, enabling malaria infection screening and five *Plasmodium* species genotyping in parallel by integrating RPA and CRISPR-based detection within a microfluidic chip [3]. Chen et al. established an integrated microfluidic detection platform Dynamic confined-space-implemented One-pot RPA-LAMP colorimetric detection system (DORLA), which combines microfluidics chip with isothermal amplification assays, offering a versatile and reliable solution for simultaneous detection of HuNoV subtypes GI and GII [5]. However, the combination of microfluidics with isothermal amplification and CRISPR has not yet been applied to detection of plant viruses.

**Fig. 1:**
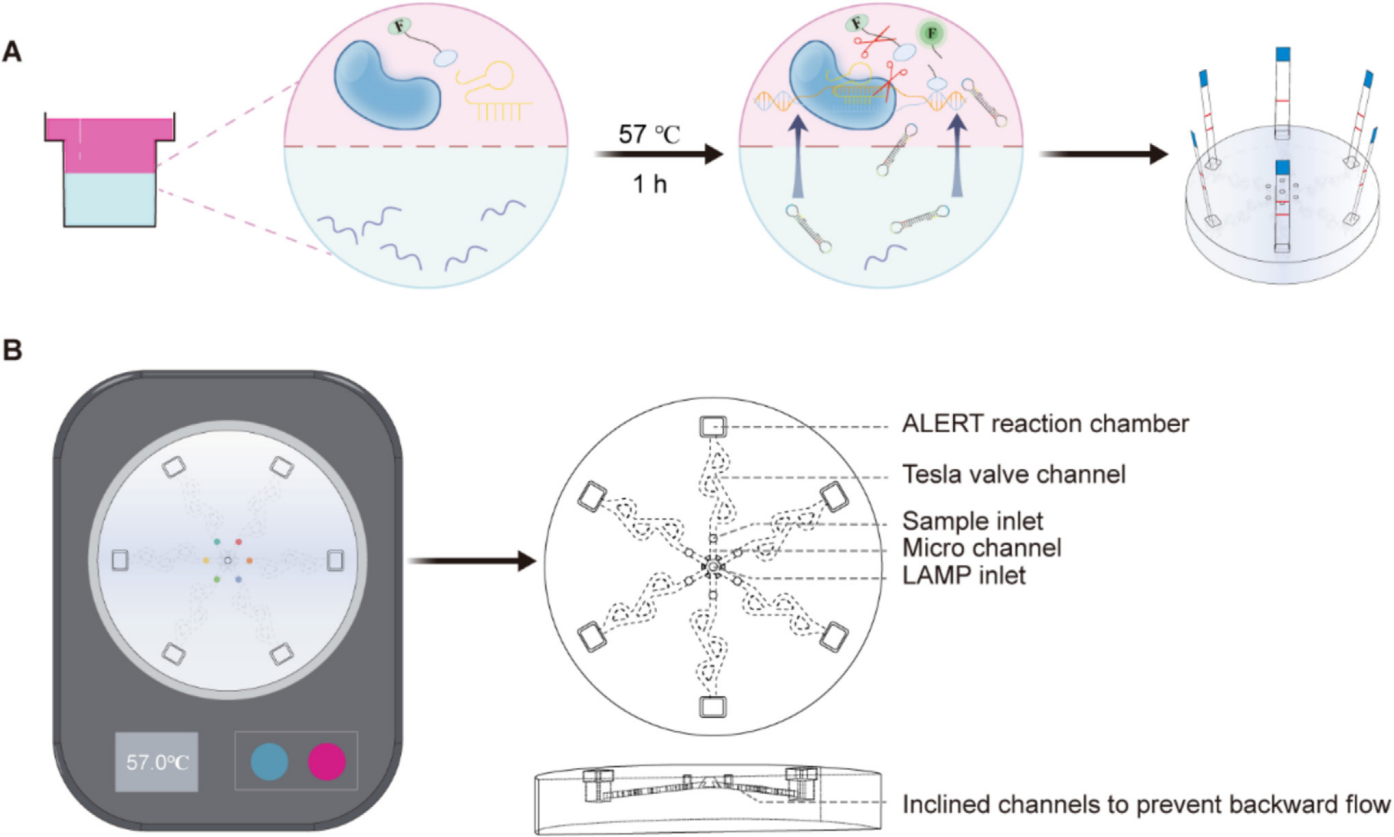
Design of ALERT platform. (**A**) Schematic overview of workflow for detection in ALERT assay. (**B**) The diagram of an incubator and a fully integrated microfluidic chip designed for the portable ALERT platform.

Here, we report an ALERT platform that combines LAMP and CRISPR-associated (Cas) 12b within a microfluidic chip for multiplexed detection of plant viruses (Fig. 1). With the assistance of the raffinose solution, an efficient one-pot LAMP-CRISPR/Cas12b system with ultrahigh sensitivity can be achieved. By integrating with microfluidic technology and lateral flow strips, we enabled simultaneous detection of six targets and visual readout. In addition, we developed a fast RNA extraction approach free of any devices at room temperature, as well as a portable incubator powered by a power bank that provides accurate temperature at 57 °C and that is applicable for LAMP-CRISPR/Cas12b reactions, making ALERT system suitable for plant viruses detection in the field. Therefore, the ALERT platform is an invaluable tool for field detection of plant viruses and shows great potential for quarantine virus detection.

## Materials and methods

### Plant and virus materials

*Nicotiana benthamiana* and pepper (*Capsicum annuum* L.) plants were grown in environmental chambers under 16-h light/8-h dark photoperiods at 25 °C. Pepper samples infected with beet western yellows virus (BWYV) were collected from fields in Dingxi City of Gansu Province (China) and used as a primary source of the virus. Field samples suspected of BWYV infection (n = 24) were collected from six regions in Gansu Province, China: Dingxi, Lanzhou, Wuwei, Baiyin, Tianshui, and Pinliang. For cucumber mosaic virus (CMV, Accession: KX883819) and pepper cryptic virus 2 (PCV-2, Accession: KT931616) detection, their respective coat protein (CP) gene fragments were cloned into the pGEM-T Easy vector (Promega, USA) to generate plasmid templates. *Agrobacterium tumefaciens*-mediated citrus leaf blotch virus (CLBV) inoculation was performed as previously described [19].

### Total RNA extraction, RT-PCR and qRT-PCR

Total RNAs were isolated from plant leaves using Trizol (Invitrogen) and first-strand cDNA was synthesized with PrimeScript™ RT reagent Kit (Takara). For rapid RNA extraction in the field, fresh plant leaves were collected and ground with lysis buffer (800 mM guanidine hydrochloride, 50 mM Tris pH 8, 0.5 % Triton X100, 1 % Tween-20) in the pouch. A silica membrane was inserted into the pouch and RT-PCR was carried out using 2 × Taq Plus MasterMix (CWBIO, Beijing, China). qRT-PCR reaction in the 25  μL mixture containing 3 μL of cDNA, 12.5 μL of Premix Ex Taq (RR390A, Takara, Japan), 0.2 μL of probe, and 0.5 μL of each primer on the Applied Biosystems QuantStudio Real-Time Quantitative PCR Instrument (Thermo Fisher Technology Co., Ltd, Shanghai, China). The amplification program was 95 °C for 5  s, 40 cycles at 95 °C for 5  s and 60 °C for 30  s. The standard curve was constructed by plotting threshold cycle (Cq) values against the logarithm of the DNA copy number.

### LAMP primers, reporter probes and crRNAs design

A set of LAMP primers were designed by the LAMP primer design software Primer Explorer version 5 (http://primerexplorer.jp/e/). To assess the specificity of designed primers, homology search was performed by nucleotide BLAST. The Cas12b/crRNAs targets the LAMP amplification region containing a specific nucleotide sequence (TTTN) called the protospacer adjacent motif (PAM) and designed following the common principles (https://www.tolobio.com/product_details/32118_AapCas12b_(C2c1)_Nuclease.html). The sequence of primers and crRNAs are outlined in Supplementary Data1. All LAMP primers, crRNAs, FQ reporter (fluorescent quenched labeled reporter: FAM-TTTTTT-BHQ1) and FB reporter (FAM-Biotin labeled reporter: FAM-TTTTTT-biotin) were synthesized by TSINGKE (Beijing, China) (Table. S1).

### Raffinose-assisted one-pot detection assay

In the ALERT system, the compatibility issue of LAMP and CRISPR were resolved through dynamic diffusion. WarmStart® Multi-Purpose LAMP/RT-LAMP 2X Master Mix (with UDG) for LAMP reaction was purchased from New England BioLabs (Ipswich, MA). *Alicyclobacillus acidiphilus* Cas12b (AapCas12b) was purchased from editgene (GuangZhou Editgene Co., Ltd, China). High-density LAMP detection mixture (bottom phase) contained 10 μM primer mix, raffinose and targets. Low-density CRISPR-Cas12b reaction solution (top phase) contained 5 μM AapCas12b nuclease, 500 nM crRNA, 4 μM ssDNA reporter and 10 × cleavage buffer. The LAMP/CRISPR-Cas12b reaction system was incubated at 57 °C, 59 °C, 61 °C, 63 °C or 65 °C, and real-time fluorescence was monitored by Microplate System. Images of all samples under blue light are recorded by the smartphone camera.

### The specificity and sensitivity of ALERT platform

To test the specificity of ALERT, plasmids containing BWYV, CMV, or PCV-2 sequences served as templates in the ALERT reaction system with BWYV-specific LAMP primers and crRNA. To evaluate the sensitivity of ALERT, different concentrations of BWYV RNA (ranging from 10^5^ to 10^0^ fg/μL) was added into the ALERT reaction system. After incubating at 57 ℃ for 1 h, fluorescence signals and lateral flow strips results were recorded.

### Design of microfluidic chip and the portable incubator

The microfluidic chip (diameter 60 mm, height 10.2 mm) was designed using SOLIDWORKS 2020 software. Microfluidic operation employed pressure-driven flow through 1 mm-diameter radial channels patterned with Tesla valves (curvature radius = 2 mm), featuring an 8° descending slope to enhance flow diffusion and prevent backflow. After fabricating with a 3D printer (UnionTech Co., Ltd, China) using clear methacrylate-based resin R4600 (WeNext Technology Co., Ltd, China), the chip was washed with isopropanol and deionized water for 15 min under ultrasonic conditions to remove uncured resin. Then 2 % polyethylene glycol (PEG) 3350 aqueous solution was statically coated onto the chip at room temperature for 30 min to improve the biocompatibility [3,20]. Finally, raffinose solution and dye solution were added to chambers for determining whether solution flow from the central inlet port to the peripheral reaction chambers successfully.

The portable incubator incorporates a precision heating system with a controllable temperature range of 10–60 °C, achieving stabilized thermal performance at 57 °C ± 3 °C. The heating core consists of a silicone thermal pad (with an integrated temperature sensor on its rear surface), regulated by the motherboard via a threshold-based control algorithm. Power is supplied through a dedicated Type-C decoy port, requiring a 5 V input with ≥ 3 A current for optimal operation. This configuration delivers a consistent heating power of ∼ 15 W during active phases, while maintaining standby power consumption below 0.5 W. User interface includes a digital temperature display and dual-button control (blue/red) for temperature adjustment, with support for rapid charging through power bank connectivity.

### LAMP and Cas12b detection on microfluidic chip

Initially, the RNA sample was introduced into sample loading chambers. Raffinose and LAMP amplification system were mixed and added into inlet port, promoting RNA sample to gather at reaction chambers, and then crRNA and CRISPR-Cas12b reaction solution was added to the top phase of LAMP system. After incubating at 57 ℃ for 1 h on a thermostatic device, test products were detected with nucleic acid stripes. Both the test line (T) and the control line (C) or only T line showed red bands in the positive result, while only C line appeared as red band in the negative result.

### Statistical analysis

The experiment was performed with three technical replicates, one biological replicate. Student’s *t*-test and one-way ANOVA were conducted to determine statistical differences between two groups or among multiple groups, respectively (*P < 0.05) using GraphPad Prism 8.

## Results

### Overview of the ALERT platform

We established a complete integrated microfluidic platform (ALERT) for multiplexed detection of plant viruses. In order for ALERT platform to be feasible for field detection, we established a portable ALERT platform consisting of a portable incubator and a microfluidic chip. To improve the sensitivity and specificity of molecular diagnosis, we combined LAMP reaction with CRISPR in the same tube (Fig. 1A). Importantly, raffinose-assisted one-pot assay design enables spatial separation of two reactions and reduced risk of aerosol contamination. After loading the sample RNA, LAMP reagents and 5 % raffinose were added to inlet port, mixed with sample RNA through micro channels and Tesla valves, arrived evenly at bottom phase of peripheral reaction chambers. Next, CRISPR/Cas12b was introduced into the reaction chambers. The microfluidic chip was placed in the incubator at 57 ℃ for 1 h. When the reaction ended, Cas12 nucleic acid lateral flow strip was inserted to detect virus.

To achieve multiplexed diagnosis simultaneously, the microfluidic chip from the center to the periphery (diameter 60 mm, height 10.2 mm) consisted of one LAMP component inlet port (diameter 1.51 mm, height 2.5 mm), six mixing micro chambers, six targets sample loading chambers, six Tesla valves for avoiding backflow and six reaction chambers for multiple Cas12b detection (Fig. 1B). Each peripheral reaction chamber comprises two linked parts: the top chamber (length 4.6 mm, width 3.6 mm and height 2 mm) and the bottom chamber (length 3.7 mm, width 3.6 mm and height 3.7 mm). The incubator provides accurate and stable temperature for reaction (external and internal structure details in Supporting Data, Fig. S1).

### Optimization of the ALERT platform

**Fig. 2:**
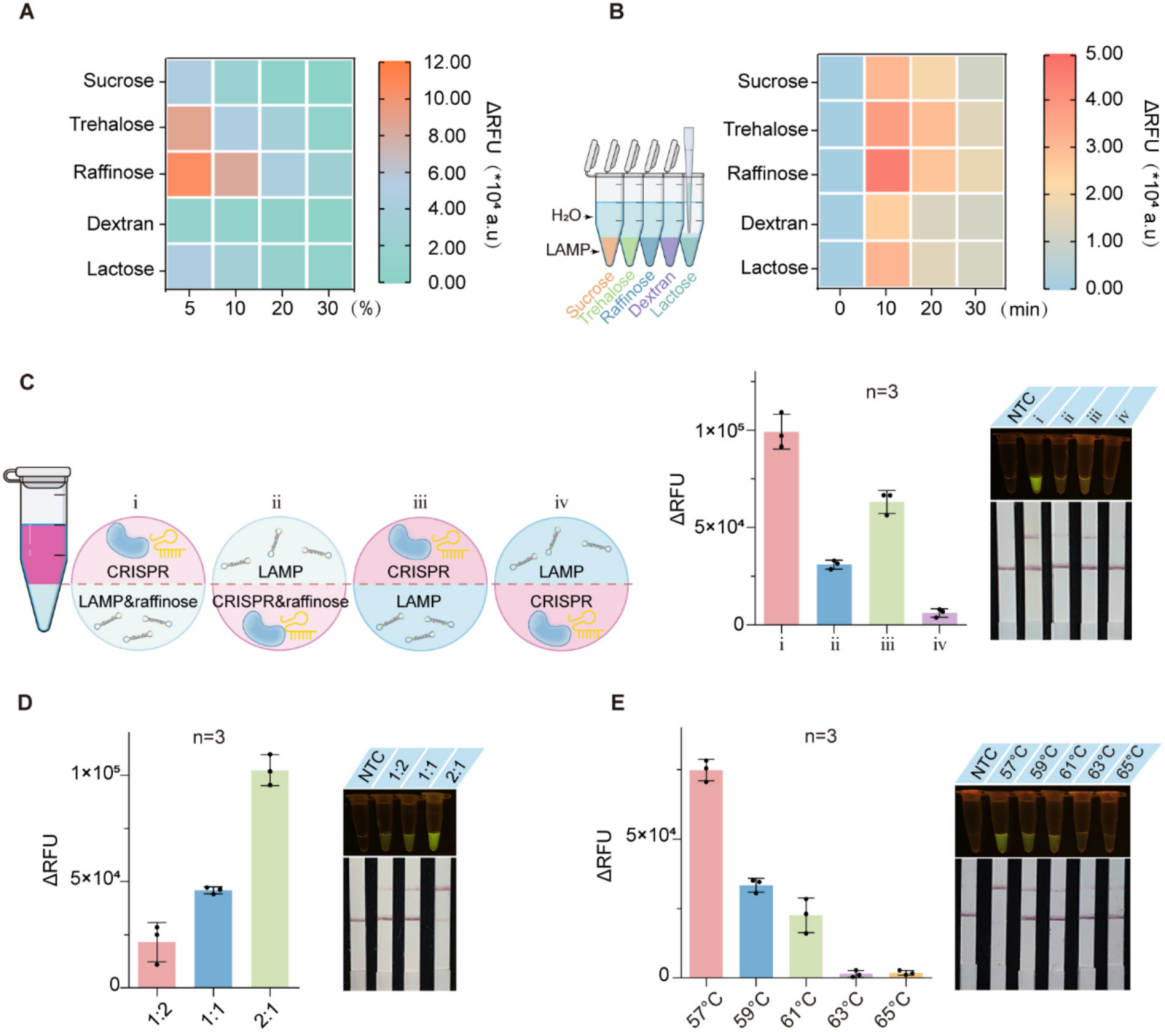
Establishment and optimization of ALERT system. (**A**) Evaluation of the effects of different sugars on ALERT reaction. (**B**) The heatmap of fluorescence signals in H_2_O phase under LAMP reaction in presence of different sugars. The values represent the groups with different sugars minus the control group (without templates). (**C**) Assessment of the effects of different addition strategies on ALERT. Group i: the LAMP reaction mixture containing raffinose was on the bottom of tube and CRISPR system was on top. Group ii: CRISPR system mixture containing raffinose was on the bottom of the tube and the LAMP was on top. Group iii: the LAMP component was on the bottom of the tube and the CRISPR system was on top. Group iv: the CRISPR system was on the bottom of the tube and the LAMP component was on top. (**D**) Fluorescence measurement comparing detection intensity of ALERT system at different volume ratio of LAMP bottom phase (5% raffinose) and CRISPR-Cas12b top phase. (**E**) Optimal working temperature for ALERT reaction efficiency.

Previous researches have demonstrated that density difference of sucrose concentration resolved the compatibility between LAMP and CRISPR to achieve spatially separated but connected phases in one-pot [3,4]. Building upon this insight, we embarked on evaluating which sugar including sucrose, trehalose, raffinose, dextran and lactose may play the most effective role in enhancing LAMP reaction component flow and detection intensity. All of these sugars facilitated the establishment of density-based phases separation (Fig. S2A). Among them, it was clearly observed that 5 % raffinose and trehalose in LAMP reaction solution both exhibited a relatively high enhancement effect in detection intensity with the highest fluorescence signal (Fig. 2A, Fig. S3). Considering cost involved, we selected raffinose as an addition and for subsequent analysis. To further explore the mechanism of raffinose-induced enhancement effect on detection performance, we first investigated the LAMP reaction activity affected by sugars. Results showed that raffinose only has little effect on LAMP activity (Fig. S4). Then we verified the dynamic diffusion between the two phases at 57 ℃. The different sugar solutions containing the dye was added to water (top phase) to form the bottom phase. However, no obvious color change visually occurred among all the treatments (Fig. S2A). Subsequently, the elevated gray areas after sugars-added for different incubation times were calculated, and a significant increase was noticed in raffinose-supplemented solution (Fig. S2B). Finally, we added 50 μL of distilled water on the top of LAMP solution (containing fluorescent dyes) with different sugars and detected amplification products in upper phase through measuring the fluorescence signal. Surprisingly, among all of these sugars, raffinose-assisted LAMP reaction displayed the most obvious fluorescent signals (Fig. 2B), indicating raffinose promoted the dynamic diffusion of LAMP products from the bottom phase to the upper phase. We further investigate detection efficiency of different addition strategies with raffinose in one-pot LAMP-CRISPR/Cas12b reaction, and the most obvious increment in the fluorescence signal was triggered by the existence of raffinose in LAMP system on the bottom of tube (Fig. 2C). In view of the above results, we optimized the volume ratio of the LAMP bottom phase (5 % raffinose) and CRISPR-Cas12b top phase. Results showed that the reaction in the volume ratio of 2:1 as chosen as the optimal working conditions for ALERT (Fig. 2D). We then explored the effect of working temperature ranging from 57 ℃ to 65 ℃ on the ALERT system. As shown in Fig. 2E, the optimal working temperature of ALERT platform is 57 ℃.

### Detection performance of the ALERT platform

**Fig. 3:**
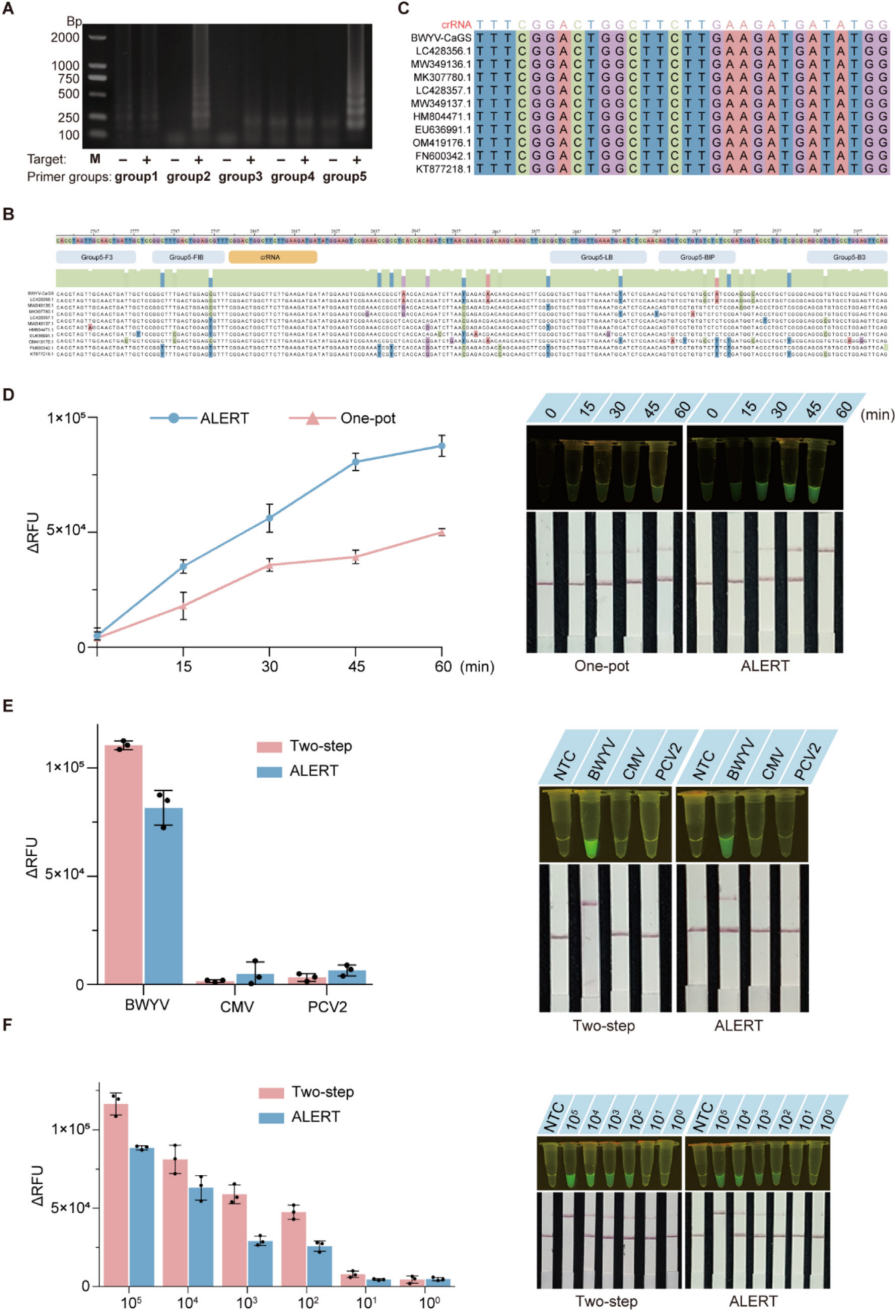
Feasibility verification of ALERT platform. (**A**) Agarose gel electrophoresis of the products for screening LAMP primer groups. (**B**) Sequence alignment for LAMP primers (highlighted in gray) and crRNA (highlighted in yellow) of eleven BWYV isolates used in the ALERT platform. (**C**) Schematic representation of different BWYV isolates sequence region targeted by the crRNA. (**D**) The performance of ALERT platform on the detection of BWYV. (**E**) The specificity of ALERT system. (**F)** Sensitivity analysis of ALERT system. NTC: no template control; CMV: cucumber mosaic virus; PCV-2: pepper cryptic virus 2; BWYV: beet western yellows virus. (For interpretation of the references to color in this figure legend, the reader is referred to the web version of this article.)

To investigate the performance of our portable ALERT platform for detecting plant viruses, five groups of primers were designed based on the highly the conserved region of RNA-dependent RNA polymerase (RdRp) of BWYV and tested to screen for the optimal primers for the LAMP reaction. Specifically, group5 mediated the strongest signal with the highest amplification efficiency (Fig. 3A) and exhibited well-conserved target sites among BWYV different isolates (Fig. 3B). Meanwhile, we designed crRNA targeting the conserved sequence based on group5 amplification products (Fig. 3C). Next, the optimal crRNA and ssDNA were investigated for the best CRISPR reaction. As shown in Fig. S5, the one-pot reaction with 150 nM crRNA and 800 nM ssDNA reached the highest fluorescence signals. Additionally, ALERT platform successfully detected BWYV with higher fluorescence intensities than the one-pot system (Fig. 3D). Notably, at the 30 min endpoint, ALERT consistently generated positive signals for BWYV, whereas conventional LAMP, RT-PCR, and qRT-PCR assays all failed to detect the virus (Fig. S6A, D, G).

To evaluate the specificity of the ALERT system, the target BWYV was detected using plant virus including BWYV, CMV or PCV-2 DNA as a template. Obviously, all tested methods generated positive signals exclusively when BWYV was present (Fig. S6B, E, H). Further, we successfully obtain a target band with BWYV in the test line using lateral flow strips. In addition, the ALERT platform displayed lower efficiencies with the two-steps system (Fig. 3E).

Next, the sensitivity of the ALERT platform was evaluated alongside LAMP, RT-PCR, and qRT-PCR using serially diluted templates. Results demonstrated that both ALERT (fluorescence-based) and RT-PCR (gel electrophoresis) detected BWYV at 100 fg/μL, while LAMP (gel electrophoresis) and qRT-PCR (fluorescence) achieved detection at 10 fg/μL (Fig. 3F, Fig. S6C, F, I). Additionally, the fluorescence signals produced by ALERT system is slightly weaker than that by two-steps system (Fig. 3F).

### Field sample testing of the ALERT platform

**Fig. 4:**
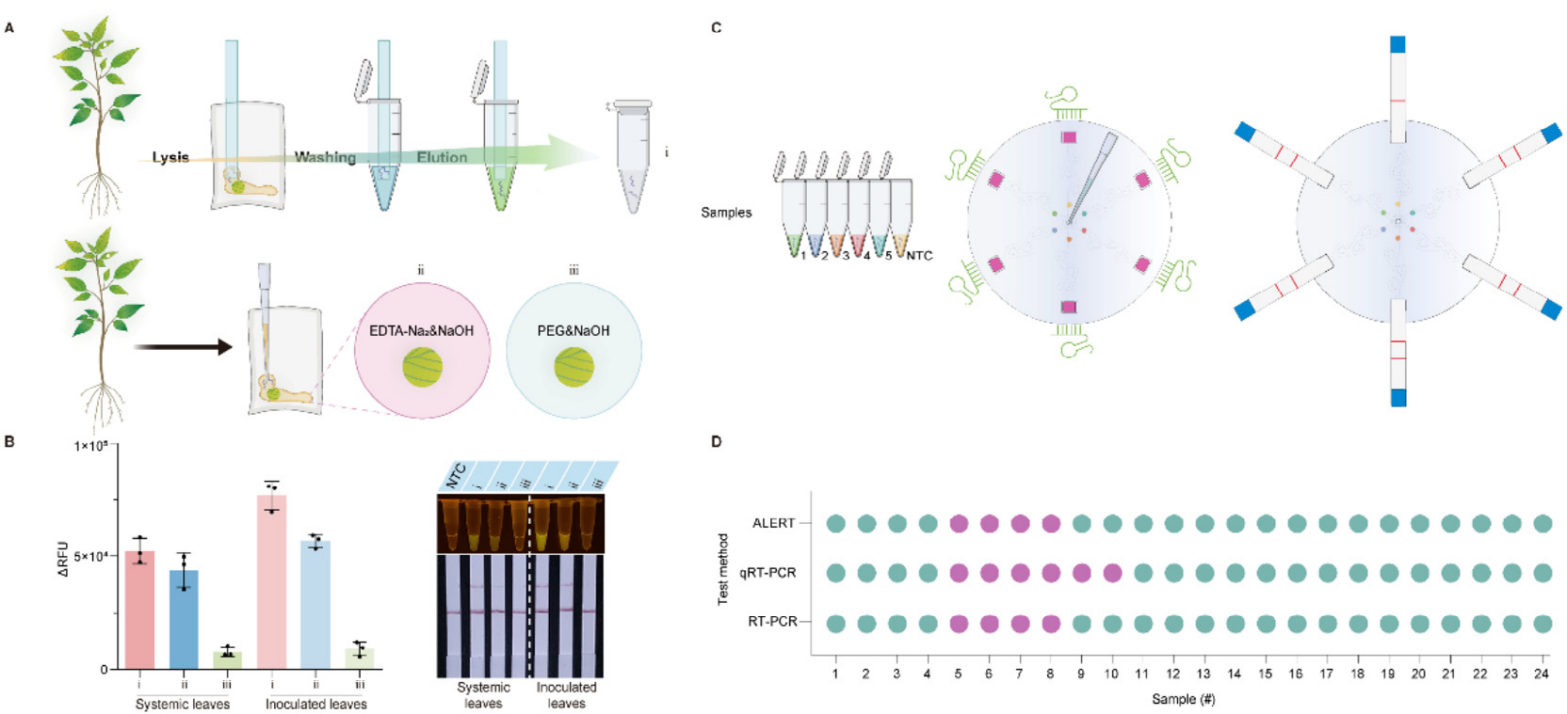
Field validation of the ALERT platform using virus-infected leaves. (**A**) An outline of the silica membrane dipstick-based nucleic acid purification method. Plant tissue was ground within lysis buffer. The silica membrane dipstick was used to capture nucleic acids by dipping it into homogenate, then dipped into wash buffer and finally transferred to a tube containing elution buffer (i). Plant tissue was ground using EDTA-Na_2_&NaOH (ii) or PEG&NaOH (iii). (**B**) Fluorescence analysis of systemic and inoculated leaves using three different nucleic acid purification methods. (**C**) Overview of ALERT platform workflow on microfluidic chip. (**D**) Horizontal comparison among ALERT, qRT-PCR and RT-PCR.

To make ALERT platform successfully achieve field-applicability, we developed a new approach to simplify nucleic acid extraction (Fig. 4A). After plant leaf tissue was ground with lysis buffer, the silica membrane was directly inserted into the homogenate to bind nucleic acids. The silica membrane dipstick was washed in wash buffer, and then transferred into a tube containing elution buffer (i). By comparing fluorescence intensities with two reported RNA extraction methods (ii, iii) [21,22], it was clearly indicated that using silica membrane dipstick (i) achieve the highest intensity in systemic leaves and inoculated leaves, consistent with that of the lateral flow strip results (Fig. 4B). Field validation was conducted on 24 pepper leaf samples (4 per region) collected from six agricultural regions in Gansu Province, China (Dingxi, Lanzhou, Wuwei, Baiyin, Tianshui, Pinliang). To streamline field operations, a microfluidic chip enabling simultaneous detection of six samples was deployed for virus identification (Fig. 4C, Video S1). BWYV was detected in Dingxi and Pingliang samples (6/24, 25 % prevalence), indicating localized outbreaks. Remarkably, our ALERT platform exhibited the same sensitivity as that in RT-PCR method, slightly lower than that in qRT-PCR (Fig. 4D, Fig. S7, Fig. S8). To assess the broad applicability of the ALERT platform, we evaluated its performance across diverse viral pathogens from distinct hosts with varied inoculation methods. First, the LAMP-CRISPR12b-based detection system for CLBV has been established (Fig. S9A, B). Then, CLBV was introduced into *N. benthamiana* via *Agrobacterium tumefaciens*-mediated infiltration. From 3 to 10 days post-inoculation (dpi), systemic leaves were collected daily from five individual plants. These samples underwent parallel analysis using both ALERT and conventional RT-PCR. Both methods consistently detected CLBV in systemic leaves at 10 dpi (Fig. S9C, D), demonstrating equivalent early-detection capability.

## Discussion

Rapid and convenient field identification of viral diseases is essential for effective disease management and eradication. An ideal diagnostic method in the field would have ability to simultaneously test multiple samples, while no such test currently exists. In this study, we presented ALERT, a multiplexed and microfluidic diagnostic platform for virus detection that can be parallelized to test six samples. By leveraging raffinose-assisted system, we overcame compatibility and contamination risks, achieving spatial separation of LAMP and CRISPR reactions. The combination of LAMP-CRISPR/Cas12b process in microfluidic chip with lateral flow strip simplifies the diagnostic process but also ensures high efficiency.

Several nucleic acid isothermal amplification detection methods (such as LAMP and RPA/RAA) have been applied to CRISPR/Cas assays to detect plant viruses [2,23,24]. The RPA/RAA technique induces non-specific detection of closely-related species due to spurious amplifications [25]. Thus, we decided to combine LAMP and Cas12b assays in one-pot for plant viral detection. The recommended temperature of LAMP reaction is 60–65 °C [6] and AapCas12b system maintains optimal nuclease activity between 31°C and 59 °C [26]. While we found the most suitable temperature of ALERT platform is 57 °C, lower than that of LAMP system, speculating Cas12b is the determining factor of amplification efficiency in ALERT platform. In the future, enhancing thermostability of Cas enzymes through engineering AapCas12b is necessary to propel ALERT platform detection to a higher level.

Multiplex capacity is critical for the molecular identification of pathogens owing to complexity of RNA isolation from multiple plant tissue samples in the field [27]. The microfluidic platform that integrates target amplification and CRISPR detection on a minimized chip, enabling contamination-free detection for diverse samples [28]. Reaction solution flows can be manipulated in microfluidic devices through driving forces such as pumps, centrifugal and capillary [29]. The pump force-driven CRISPR microfluidic chip comprising multiple reservoirs can detect 24 Ebola samples in parallel practically within 30 min [30]. By combining RAA with CRISPR-Cas12a system, the centrifugal microfluidic chip was developed to simultaneously detect 16 samples [28]. For the detection of plant viruses, the platform integrating multiplex RT-RPA and separate LbCas12a enables simultaneous detection of five prevalent apple RNA viruses/viroid-apple necrotic mosaic virus (ApNMV), apple stem pitting virus (ASPV), apple stem grooving virus (ASGV), apple chlorotic leaf spot virus (ACLSV), and apple scar skin viroid (ASSVd), but requires a two-step procedure involving post-amplification transfer to separate Cas12a/crRNA reaction tubes, which increases risks of aerosol contamination and operational complexity [31]. Subsequently, a one-tube RPA/RAA-Cas12a platform was developed for simultaneous identification of pepper mild mosaic virus (PMMoV), tomato brown rugose fruit virus (ToBRFV), tomato mosaic virus (ToMV), and tomato mosaic virus (ToMMV) in infected tomato and pepper plants, using a generic RT-RAA primer set combined with four target-specific crRNAs [7]. However, in one-pot assays, the activated *trans*-cleavage activity of CRISPR-Cas12b reduces primer concentrations, leading to decreased sensitivity. Compared with previously reported microfluidic platform, we designed a microfluidic device driven by pipette-produced pump to achieve multiplex detection, controlled by a single inlet. Utilization of Tesla valves can effectively prevent backflow of reaction solution, then avoid contamination of different plant RNA samples. Additionally, identification results can be easily obtained through lateral flow readouts, achieving visual detection for plant viruses. Crucially, our comparative experiments results prompted the testing of the feasibility and accuracy of the ALERT platform in field applications.

Dozens of isothermal amplification-Cas12 systems have been developed for plant viral detection, while the conventional approaches typically involve a two-step process [32] or an all-in-one reaction regardless of biocompatibility issues [7]. Sucrose enhances dynamic molecular diffusion by forming hydrogen bond network (HBN) [5]. Recently, the inclusion of 10 % sucrose solution effectively addresses the compatibility issues associated with LAMP and CRISPR assays within a one-pot system, simplifying the workflow and ensuring high efficiency for *Plasmodium* species screening [3]. In the present study, the introduction of raffinose exhibited the highest amplification efficiency among five different sugars, and 5 % raffinose provided the best detection performance when incorporating into LAMP system at the bottom (Fig. 2A, C). Moreover, raffinose-assisted one-pot reaction showed high sensitivity approaching two-step system for detection of BWYV (Fig. 3F), indicating ALERT system successfully solves the poor compatibility between LAMP and CRISPR reactions without compromising the sensitivity, and reduces carryover contaminations due to multiple liquid-handling steps. While sucrose, trehalose and raffinose all stabilize biological enzymes [33], raffinose achieves significantly enhanced dynamic diffusion of LAMP amplicons toward CRISPR components in our assay (Fig. 2B; Fig. S2).

The raffinose-assisted one-pot assay containing 15 μL volume of the LAMP reagent and 30 μL volume of CRISPR reagent for simultaneous six samples detection, the cost for per sample was roughly 6.14 $. The resin cost for a 3D-printed microfluidic chip was 1.95 $, leading to total cost at 8.09 $ per assay. Further, the consumption of test strips for signal readout increased to 12.06 $, which is affordable in resource-limited settings. In addition, ALERT platform reduces detection time (less than 1 h) through achieving the amplification and detection in a single chamber, and without complicated RNA extraction steps. Given these excellent advantages, the ALERT system has high potential for application to field plant virus detection. When extending ALERT platform to other viruses, two critical factors must be addressed: (1) rigorous screening of LAMP primers to achieve both high specificity and maximal amplification efficiency; (2) 57 °C is universally optimal for all tested viruses, eliminating temperature screening needs, while increasing temperatures invariably compromise detection efficiency. The platform's user-friendly workflow, requiring only simple pipetting steps and minimal instrumentation, further enhances its practicality for field use and ensures rapid adoption with minimal training. This capability empowers timely decision-making and facilitates critical early interventions like precision control measures. By preventing localized outbreaks from escalating into devastating epidemics, particularly in resource-limited settings lacking traditional lab access, ALERT significantly minimizes yield losses and reduces reliance on broad-spectrum pesticides. Importantly, ALERT platform holds promising potential for detecting pathogens. However, the most significant challenge lies in optimizing efficient DNA extraction methods from pathogenic fungi/oomycete, which often exhibit rigid cell walls and inhibitory compounds.

Field deployment of the ALERT platform currently faces two primary environmental challenges: sample degradation during summer collection at elevated temperatures and interference from sand/dust affecting microfluidic chip operation. For future implementation aimed at enhanced robustness and wider applicability, we propose integrating the entire chip-heater assembly into a fully-enclosed device. This enclosure would effectively eliminate environmental interference from airborne particulates. Furthermore, incorporating active cooling capability into the integrated thermal control module within this enclosed system represents a promising avenue to directly mitigate sample degradation issues encountered under high ambient field temperatures.

## Compliance with ethics requirements

This article does not contain any studies with human or animal subjects.

## CRediT authorship contribution statement

**Jiahui Wang:** Conceptualization, Formal analysis, Data curation, Writing – original draft. **Wenqing Yang:** Methodology, Software, Investigation. **Jingjing Fang:** Software. **Henghao Xu:** Methodology. **Jianyao Li:** Methodology. **Yibin Zhang:** Visualization. **Qiaolan Liang:** Supervision. **Bingliang Xu:** Supervision. **Huixia Li:** Supervision. **Erbo Niu:** Conceptualization, Methodology, Validation, Formal analysis, Writing – review & editing, Visualization, Project administration, Funding acquisition.

## Declaration of competing interest

The authors declare that they have no known competing financial interests or personal relationships that could have appeared to influence the work reported in this paper.

## Data Availability

All data supporting the findings of this study are available within the paper and its Supplementary Information files.

## Appendix A Supplementary material

Supplementary Data 2

Supplementary video 3 A video of the liquid flow on the microfluidics chip
