# Supplementary material for: Field-parallel six-sample microfluidic detection of plant viruses via raffinose-assisted one-pot LAMP-CRISPR/Cas12b
Source: J Adv Res. 2025 Aug 22;83:81–9. doi: 10.1016/j.jare.2025.08.030 (PMC13131449; doi:10.1016/j.jare.2025.08.030)
Supplement: Supplementary Data 1 [file mmc1.docx]

**Field-parallel six-sample microfluidic detection of plant viruses via raffinose-assisted one-pot LAMP-CRISPR/Cas12b**

Wang *et al*


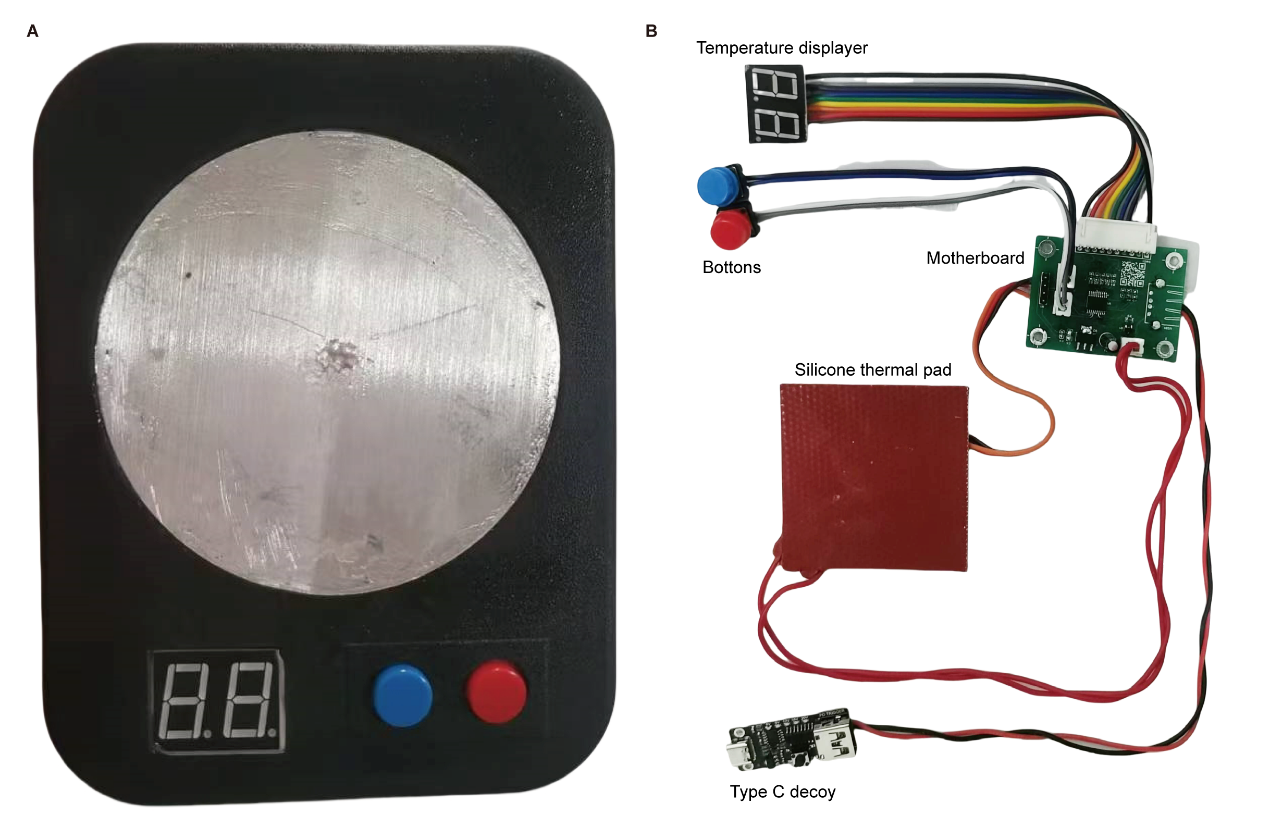


**Figure S1** Diagram of the portable incubator. (A) Photograph of the device from top view. (B) Schematic of the interior design of the device.


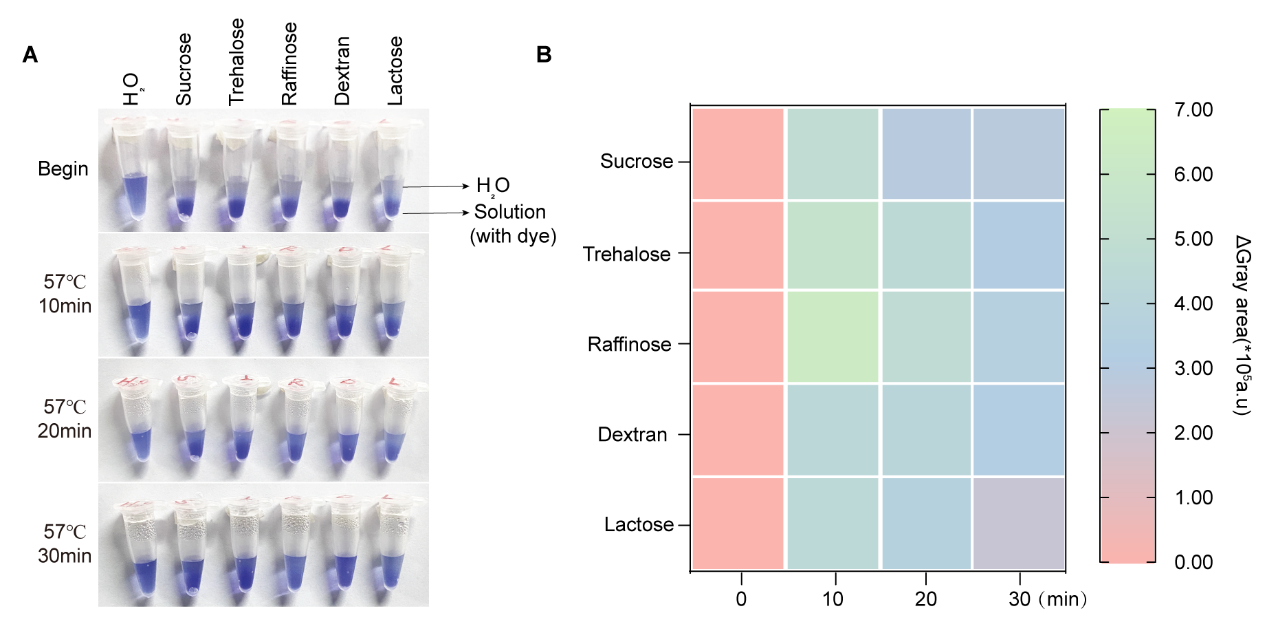


**Figure S2** The dynamic diffusion of various sugar solutions under different incubation times at 57 ℃. (A) Visual observation of dye diffusion. (B) Heatmap of evaluated gray areas supplemented with different sugars. The gray areas without incubation (0 min) were used as control.


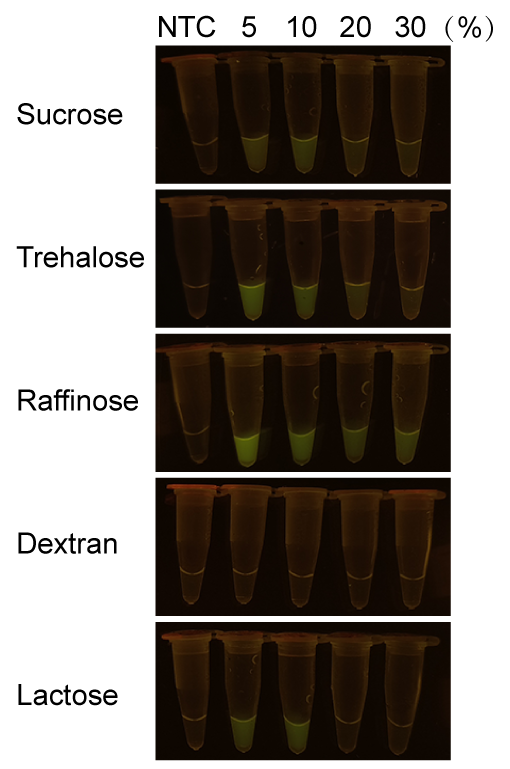


**Figure S3** Visualization of fluorescence produced by ALERT reaction with various concentrations (5%, 10%, 20% and 30%) of different additives. NTC represents no additives control.


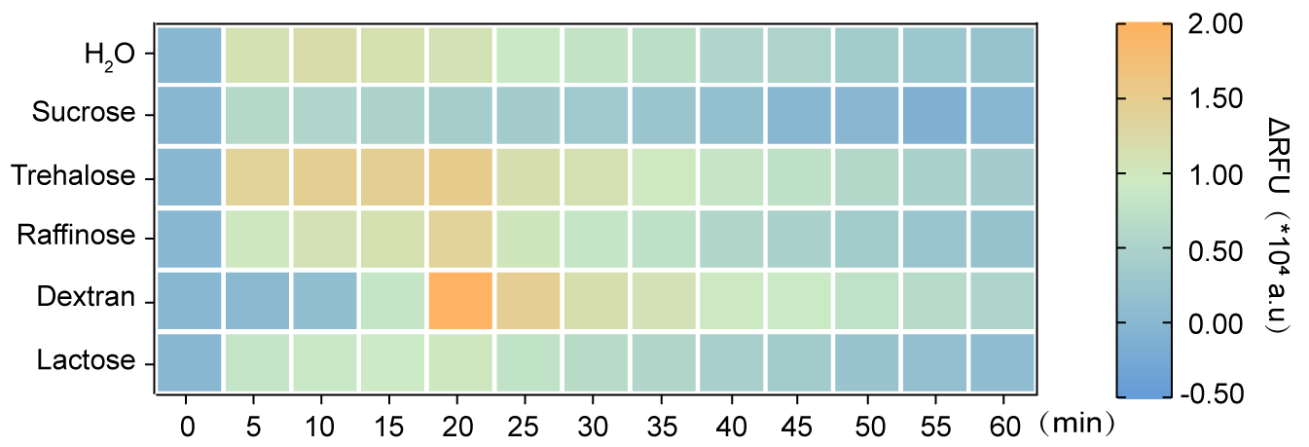


**Figure S4** Evaluation of different sugars on LAMP reaction activity.


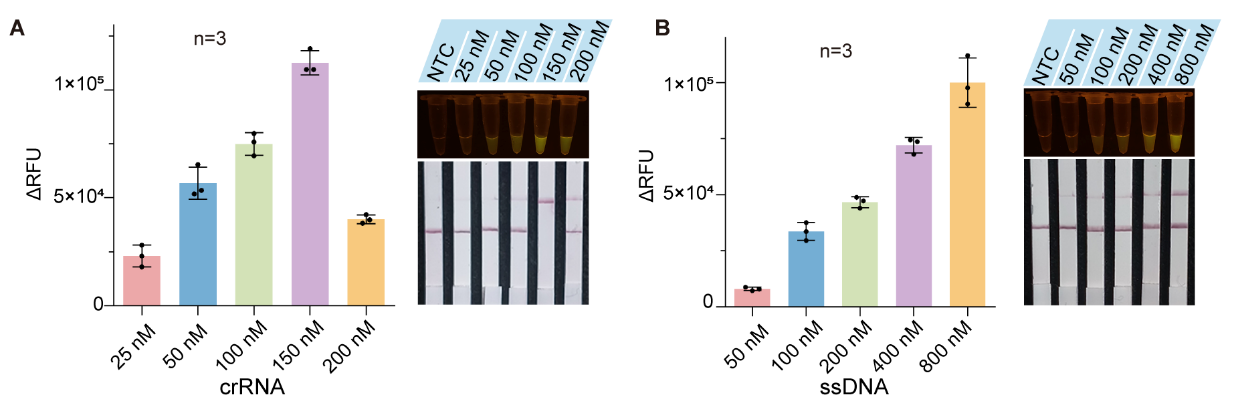


**Figure S5** Comparison of fluorescent signals of ALERT reaction at different crRNA (A) and ssDNA (B) concentrations.


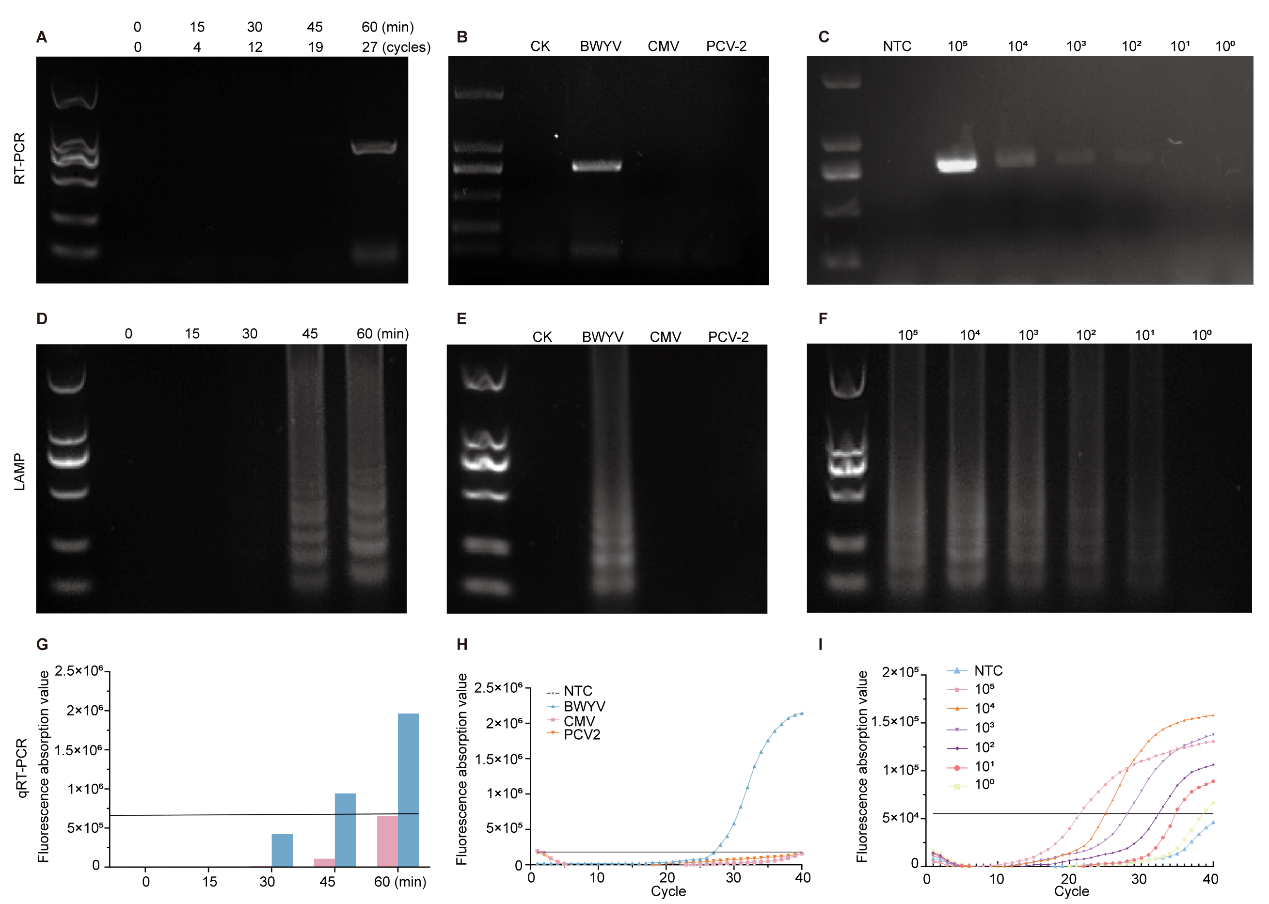


**Figure S6** Comparative performance of RT-PCR, LAMP, and qRT-PCR. NTC represents no template control. Top row (A-C): RT-PCR; Middle row (D-F): LAMP; Bottom row (G-I): qRT-PCR. (A, D, G) Time-course detection; (B, E, H) Specificity assessment; (C, F, I) Sensitivity assessment.


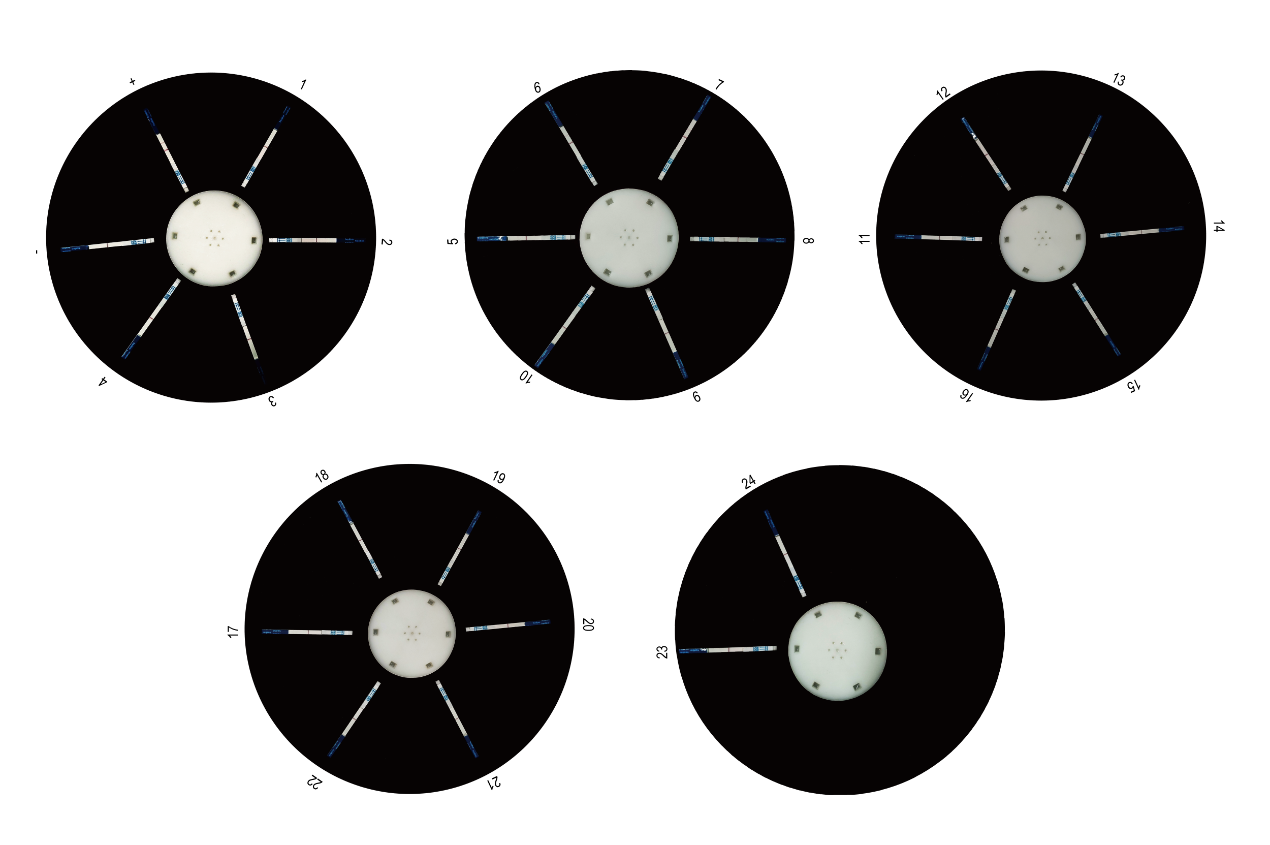


**Figure S7** Field detection of twenty-four samples in ALERT platform.


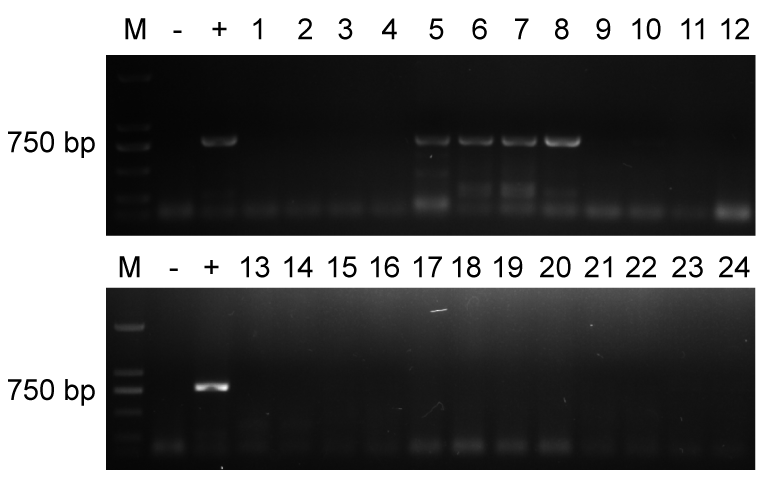


**Figure S8** RT-PCR detection of twenty-four samples in the field.

**
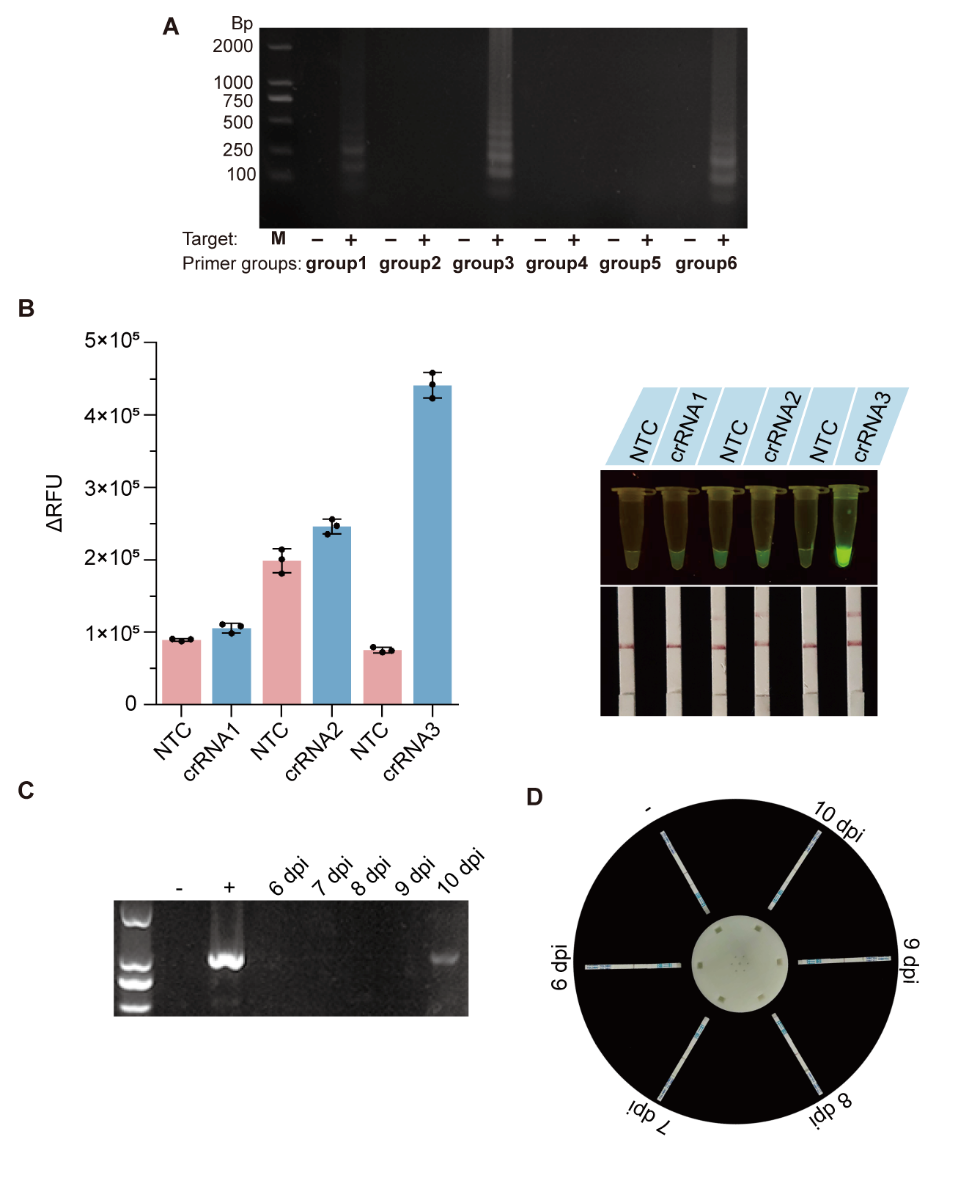
**

**Figure S9** CLBV detection using the ALERT platform. (A) Screening of CLBV LAMP primers. (B) Evaluation of CLBV crRNAs. Time-course analysis of CLBV in systemic *N. benthamiana* leaves by RT-PCR (C) and ALERT platform (D).
